# Development of a monitoring system for disassembled towers with internal suspension poles

**DOI:** 10.1038/s41598-022-21395-x

**Published:** 2022-10-07

**Authors:** Long-Bin Zhang, Bo Tang, Kai Li, Zhi-Yu Shang, Yue Wang, Heng-Bo Li

**Affiliations:** 1 College of Electrical Engineering & New Energy, China Three Gorges University, Yichang, China; 2 Hubei Provincial Engineering Technology Research Center for Power Transmission Line, Yichang, China; 3 Anhui Transmission and Distribution Engineering Co.Ltd, Hefei, China

**Keywords:** Energy science and technology, Engineering

## Abstract

The traditional construction monitoring methods of suspended pole-mounted decomposed towers are mostly manual monitoring. The monitoring personnel has multiple blind spots, and the possibility of misjudgment based on personal experience is relatively large. It is difficult to ensure the construction safety of the suspended pole decomposing tower. For this reason, combined with the current power Internet of Things technology, this paper develops an intelligent monitoring system for suspended pole-mounted decomposing towers. According to the construction technology and its safety requirements of inner suspension derrick for transmission tower erection in sections, this system is classified into intellisense layer, wireless transport layer and information integration layer. According to the physical characteristics of the seven major risk points of the inner suspension pole group tower, the intellisense layer developed corresponding sensing equipment to obtain risk information. In the wireless transport layer, the ZigBee and 4G communication technologies are selected to interconnect self-constituted LAN and 4G wide area networks, to complete on-site data interaction and long-distance transmission. In the information integration layer, the force of cable, the inclination and height of derrick, and the distance between derrick and tower are determined. The system has been verified by the 500 kV delivery project of Fujian Zhouning Pumped Storage Power Station. The average error of critical monitoring point data is 4.14%, and the average data transmission delays in the far and near fields of the system are 18 ms and 176 ms.

## Introduction

With the continuous development of the construction of Ultra High Voltage (UHV) projects, the height of the transmission line towers continues to increase, resulting in a corresponding increase in the risk of tower construction^1^. When faced with tens of meters of metal derricks and ton-weight iron tower material, construction based on the experience of on-site commanders and construction personnel is easy to cause safety accidents^2^. The occurrence of the accident not only affects the progress of the expedition construction, but also seriously threatens the life safety of the construction workers. Therefore, the development of an intelligent monitoring system for tower assembly that can perceive the safety status of inner suspension derrick and iron tower material in real-time, and comprehensively gather construction information for danger warning is an urgent need to ensure the safety of UHV tower construction.

In practical engineering applications, the development of the tower assembly monitoring system was very early. For example, literature^3^ developed a set of a video surveillance system for tower construction, and for the first time installed a surveillance camera on the top of the derrick. However, a pure video surveillance system cannot obtain the core information that affects construction safety, such as the force of the ropes used for tower assembly, the posture of the derrick, and so on. In fact, it cannot solve the problem of tower construction safety. Subsequently, on the basis of video surveillance, the literature^4^ added the cable tension monitoring functions to the tower assembly monitoring system, but the system uses wired cables to transmit signals, which affects the normal tower assembly construction. Literature^5^ improved the signal transmission method of the monitoring system based on the literature^4^, and used Wi-Fi wireless communication technology to transmit on-site signals. However, due to a large number of obstructions in the field, Wi-Fi signal transmission was limited, resulting in the inability to know the dangerous situation in real-time. Literature^6^ adopts ZigBee wireless communication technology, which is more suitable for the field construction environment, but it only monitors the cable tension, and the amount of information obtained is too small to perform the role of safety monitoring.

In order to solve the problem that the monitoring parameters are one-sided and the communication mode is not suitable for engineering practice, this paper develops a set of suspended pole-mounted and disassembled group tower intelligent monitoring system based on the power Internet of Things technology. All-round monitoring of the force of the poles and hangers and the danger of their spatial posture; at the same time, the ZigBee communication network and the 4G communication network are interconnected to realize the near and long-distance visual monitoring and real-time early warning of the dangerous situation of the tower construction. The system developed in this paper makes up for the deficiencies of the existing suspended pole-mounted disassembled group tower monitoring system in terms of monitoring parameters and communication methods. The system has been verified by the 500 kV delivery project of Fujian Zhouning Pumped Storage Power Station, and can further improve the safety of the suspended pole-mounted tower.

## Methods

### System overall design plan

Inner suspension derrick for transmission tower erection in sections refers to a tower assembly method in which the derrick is placed in the centre of the tower body, and the entire derrick is in a suspended state^7^. The bottom of the suspension derrick is towed by four supporting ropes tied to the bottom of the main material, and the top of the suspension derrick is fixed by four inner (or outer) cables tied to the upper part of the main material or connected to the ground anchor. According to the suspended derrick-holding tower assembly process and the analysis of a large number of tower-building engineering accidents, the current suspended derrick-holding tower construction mainly has the following two unsafe factors:

1. The current commander’s judgment on the dangerous situation of the tower construction mainly comes from personal experience. However, due to a large number of derricks and ropes and complex forces in the suspended derrick system, it is difficult to accurately judge the stress state of the derricks and iron tower material based on experience alone.
2. The iron tower is usually tens of meters high. The commander’s sight distance will be limited when the tower is assembled to a higher section due to the complex construction environment. Manual monitoring will inevitably miss the dangerous construction points, and it has extensive security hidden danger.

In view of the above two problems in the traditional suspended derrick-holding and tower grouping method, online monitoring by wireless sensors is considered to realize all-around and full-process real-time perception of the tower construction situation. The specific monitoring requirements are as follows:

1. Force monitoring of the cable. As an important component for fixing the suspension derrick, if one of the ropes breaks due to the inability to withstand external forces, it will directly destabilize the entire derrick system, resulting in serious safety accidents such as the derrick tipping and the falling of the tower. Therefore, a tension sensor is installed on each of the four lifting ropes connected to the tower body to monitor the tension value of the cable.
2. Force monitoring of the hoisting rope. Hoisting ropes are the main force-bearing parts of lifting towers. Tonnage components need to be lifted to high altitude during work. If the force overload causes the ropes to break, it will cause the tonnage lifting components to lose stability and fall from high altitude. Therefore, one tension sensor is installed on each of the two hoisting ropes to monitor the tension value of the hoisting rope.
3. Posture monitoring of derrick holding. The derrick is an essential component in the entire tower assembly process, and the construction regulations limit its maximum inclination. Therefore, an inclination sensor is installed inside the derrick to monitor the working inclination value of the derrick. In addition, even if the inclination angle of the derrick does not exceed the specified value when the construction is carried out in some difficult terrain, the derrick may contact and collide with the tower body at this time, causing serious safety accidents such as breaking of the derrick and falling of the tower structure. Therefore, it is also necessary to monitor the distance between the derrick and the tower body.

However, considering that the derrick and the tower body are dynamic movements between the lattice members when the tower is assembled, the distance between the two is difficult to monitor in real-time by means hardware, so it is decided to derive the method of real-time calculation formula for the distance between the derrick and the tower body, to monitor the distance between the derrick and the tower in real-time.

(4) Height monitoring of derricks and iron tower material. When assembling some taller towers or tower tops, the commander under the tower cannot judge whether the tower is in place and whether the derrick needs to be lifted based on the lifting height of the tower and the working height of the derrick due to the limited viewing distance. Therefore, a height sensor is integrated on the hoisting rope sensor to measure the lifting height of the hoist; a distance measuring sensor is integrated into the derrick inclination sensor to measure the distance between the derrick and the ground to reflect the working height of the derrick in real-time.
(5) Monitoring of wind speed. Since the derrick is a slender component, the wind resistance is weak. Once the wind at the construction site is strong, major tower construction accidents such as derrick overturning may occur. The project also strictly stipulates that the wind speed is greater than level 5, and no operation is allowed. From the relationship between height and wind speed, it can be seen that the top of the derrick receives the largest wind force. Therefore, a wind speed sensor is installed on the top of the derrick to measure the real-time maximum wind force on the construction site.

**Figure 1:**
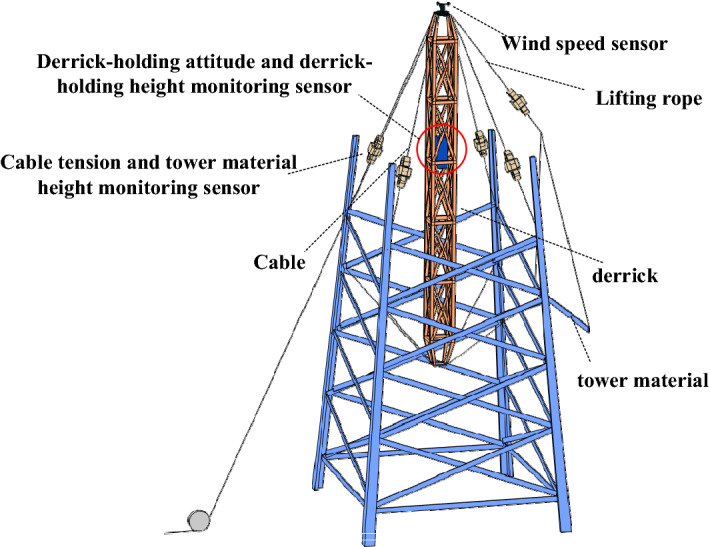
Schematic diagram of system monitoring point setting.

To sum up, the monitoring layout of the above seven risk points is shown in Fig. 1.

### The overall structure of the system

**Figure 2:**
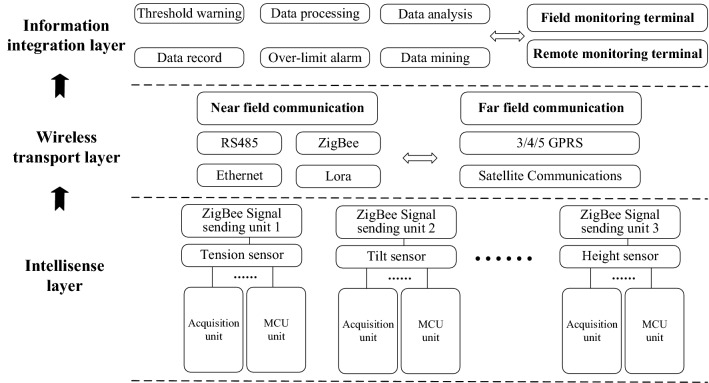
Overall structure diagram of the system.

With the development of technology, the Internet of Things in Power Systems (IOTIPS) has become an industrial-grade Internet of Things widely used in power systems due to its comprehensive state perception, efficient information processing, and convenient and flexible applications^8^. In this way, the overall framework of the system also adopts the IOTIPS framework, which is divided into three layers, namely the intellisense layer, the wireless transmission layer and the information integration layer, as shown in Fig. 2.

The intellisense layer realizes accurate collection and rapid processing of status information, which mainly includes various sensors and their built-in data acquisition units, power supply units, MCU (Microcontroller Unit) units, etc., which are used to monitor the construction process of the system Real-time collection and processing of information such as pulling force, inclination angle, and distance. The wireless transmission layer relies on various existing communication technologies to provide long-distance and short-distance transmission channels for the data collected by the intellisense layer. Therefore, RS485 serial port, Ethernet, ZigBee, Lora, GPRS and Satellite Communications can be independently selected according to the characteristics of the tower construction project, realizing the vertical information interaction between the intelligent sensing layer and the information integration layer. When designing a specific monitoring system, we need to select the most appropriate data transmission technology according to the environmental characteristics of the transmission tower construction site. The information integration layer realizes the vertical integration and horizontal integration of massive information, and makes reasonable use of the value of data, including human-computer interaction tablets and remote monitoring PC terminals, used to analyze and process on-site monitoring data, and perform threshold and over-limit data early warning or alarm, and recording the dangerous data in the background to provide support for the analysis of the force data of the tower construction and data mining.

The above-mentioned intellisense layer, wireless transmission layer and information integration layer include various hardware modules such as system collection, processing, communication, and a series of software programs that promote the operation of the system. To discuss the design ideas more clearly, the system development process will be divided into two parts: system hardware design and system software design.

### System hardware design

The wireless sensors used in the system include tension sensors, wind speed sensors, height sensors, inclination sensors and distance sensors, which are responsible for data collection and processing of the status of the installed location. According to the realization principle of sensor function, each sensor mainly contains data acquisition unit, system processing module and data communication unit. Since the internal difference of each sensor mainly lies in the form of the data acquisition unit, all sensor system processing modules and data communication units adopt a unified design concept.

The data acquisition unit of the tension sensor mainly includes strain type and piezoelectric type^9^. Because the piezoelectric sensor needs special moisture-proof measures in actual use, it cannot be used in the construction site of the iron tower with significant climate change, while the resistance strain tension sensor has high precision, good stability and strong engineering adaptability advantage.

The main forms of wind speed sensor data acquisition unit are mechanical wing type, thermal effect type and ultrasonic type^10^. The thermal effect wind speed sensor has the advantage of a wide measurement range, but it is greatly affected by the on-site humidity and temperature, so it is not considered to be used. Ultrasonic wind speed sensor has the advantages of high precision and stable performance, because of its large size and high cost, it is also unsuitable for wind speed monitoring in this system. The mechanical wing wind speed sensor meets the requirements of the tower assembly construction monitoring in terms of measurement accuracy and measurement range, and has the advantages of small size, low cost and convenient installation.

The main forms of altitude sensor data acquisition units are barometric altitude, radio altitude and GPS positioning. The radio altitude and GPS positioning monitoring methods have the advantages of high monitoring accuracy, but the complex terrain conditions of the tower assembly construction site and the many obstructions make the signal transmission difficult, and cannot be used in the tower assembly project^11^. The pressure altitude monitoring method adopts the pressure altitude sensor for monitoring, which has the characteristics of not being affected by obstacles, convenient installation and wide measurement range.

The data acquisition unit of the ranging sensor is in the form of ultrasonic and infrared ranging^12^. The ultrasonic ranging method has the advantages of good directionality and strong penetrating ability, but due to the noisy environment of the tower assembly construction site, which has a great impact on the measurement results, it is not considered to be used. The infrared ranging method has the advantages of high precision and convenient installation, but it cannot be accurately positioned during the movement of the pole, and the range will be sharply reduced in foggy days. It is also not suitable for the tower assembly project. UWB (Ultra Wide Band) ultra-wideband technology is a communication technology that relies on pulse data transmission. Due to the strong anti-interference ability of pulse signals and mobile measurement, the use of UWB technology to monitor the change of the distance between the pole and the ground can effectively avoid the measurement errors due to weather or obstructions.

The data acquisition unit forms of the tilt sensor mainly include solid pendulum, gas pendulum and liquid pendulum. The measurement accuracy of the gas pendulum inclination sensor is greatly affected by temperature and is unsuitable for field construction environments. The liquid pendulum inclination sensor is relatively fragile and easily damaged after being bumped, and it is also not suitable for the construction environment of the tower. The solid pendulum inclination sensor has a wide measurement range and good applicability in field engineering^13^.

The signal collected by the system acquisition module needs to be processed by the processing module before it can be sent to the communication module for data transmission. Therefore, it is necessary to select and design the system processing module.

The analog-to-digital conversion unit realizes the conversion of the acquired data from analog signals to digital signals, and is the key element connecting the system acquisition module and the system processing module. The Delta-Sigma analog-to-digital converter has the characteristics of fast conversion speed and low power consumption. Therefore, the form of the analog-to-digital conversion unit adopts the Delta-Sigma type, and the model adopts the ADS1232^14^.

After the signal is converted, it needs to be analyzed and processed by the MCU (Microcontroller Unit) unit^15^. The STC series has the characteristics of low power consumption and a simple program, which can better meet the data processing requirements of the system. Therefore, the system MCU unit type adopts the STC series, and the model selects STC12LE5616AD.

The working voltage of the analog-to-digital conversion unit is different from that of the MCU unit, and the two cannot be directly connected. Therefore, a level converter TXS0104EPWR is connected between the analog-to-digital conversion unit and the MCU unit to ensure the normal operation of the circuit by balancing the operating voltage between them. In addition, the 12.6 V power supply cannot directly power components of various voltage levels. Therefore, the data acquisition unit, the analog-to-digital conversion unit and the level conversion unit with an operating voltage of 5 V share the level converter LP2591AC, and the MCU unit with an operating voltage of 3.3 V uses the level converter TPS562200 to realize the normal operation of the circuit.

To prevent the signal interference generated during system operation from affecting them, and to avoid the entire circuit due to component burnout, an isolator ADuM120x is added between the MCU unit and the system communication unit.

**Figure 3:**
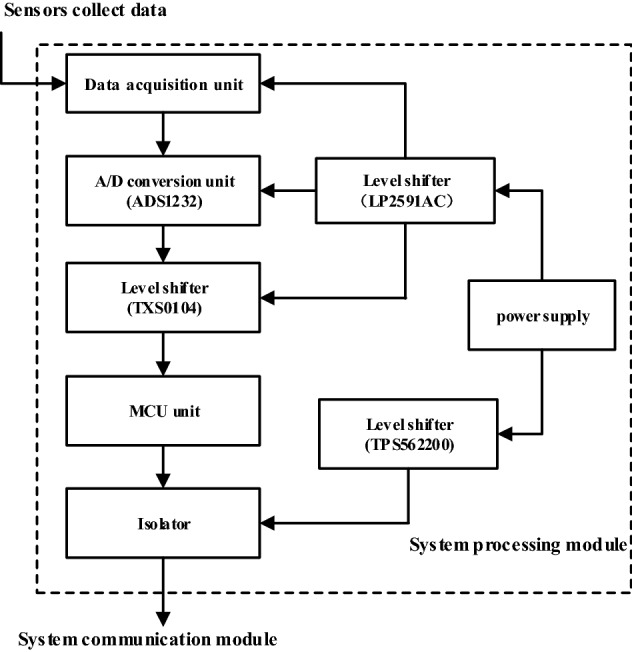
Working principle diagram of data processing unit.

To make the system complete data collection and processing, the above mentioned units need to be reasonably integrated and designed. When the system is working, the data acquisition unit first collects the monitoring data of the tower. Subsequently, the analog-to-digital conversion unit ADS1232 converts the collected electrical signals into digital signals. The MCU unit STC12LE5616AD analyzes and processes the digital signal from the analog-to-digital conversion unit and sends it to the system communication module to finally complete the entire data acquisition and processing process. Among them, the level converter TXS0104EPWR is used to balance the working voltage between the analog-to-digital conversion unit and the MCU unit, and the isolator ADuM120x is used to protect key components from burning the entire circuit. After the power supply is balanced through the level converters LP2591AC and TPS562200, the data acquisition unit, an alog-to-digital conversion unit, level converter and MCU unit are supplied with power respectively. The working principle of the system processing module is shown in Fig. 3.

The signal processed by the system processing module can only be transmitted through the system communication module to realize the signal local or remote transmission. Therefore, this section mainly selects and designs system communication modules. The choice of communication technology will directly determine the effect of data transmission in the project. The data transmission mode of this system includes on-site and remote data transmission to realize on-site of tower construction conditions and remote data sharing.

Most tower construction sites are located in remote mountainous areas, with dangerous terrain conditions and complex construction conditions. Therefore, when selecting the communication technology for on-site data transmission, it is mainly based on the following two principles:

- (1) Whether the communication technology can meet the needs of on-site construction. The site construction conditions of the tower are complex and the area is large, and the transmission distance of the communication technology should reach 100 m. At the same time, because the tower height can reach 100 m, it is difficult to replace the sensor battery frequently, and the power consumption of the communication technology should be low.
- (2) Whether the communication technology can meet the demand for a timely warning. Since the system is mainly used for the real-time perception of the stress of each key point of the tower system and early warning, the transmission delay of the communication technology should reach the millisecond level to meet the system’s timely response to dangerous situations.

Therefore, the determination of communication technology should also consider indicators such as transmission distance, power consumption, and transmission delay. Currently, the wireless communication technologies used in field data transmission mainly include Bluetooth (Bluetooth), Wi-Fi (Wireless Fidelity), Lora (Long Range Radio) and ZigBee, etc. The detailed performance comparison is shown in Table 1.

**Table 1:** Performance comparison of various communication modes.

Communication mode	Main frequency band	Transmission distance	Transmission delay	Power consumption
Bluetooth	2.4 GHz	Dozens of meters	Second level	Normal
Wi-Fi	2.4 GHz	Hundred meters	Millisecond level	High
Lora	470 ~ 510 MHz	Up to 20 km in the suburbs	Second level	Very low
ZigBee	2.4 GHz	Up to 2 km in the suburbs	Millisecond level	Low

It can be seen from Table 1 that the usual transmission distance of Bluetooth is only a few tens of meters, so it is not considered to be used; the characteristics of Wi-Fi devices that consume too much power and require frequent charging are also not suitable for the network of this system; the transmission delay of Lora is usually several About seconds, although it can be applied to the fault location of distribution lines^16^, it is difficult to meet the real-time requirements of the tower construction site. ZigBee is a medium-to-long-distance, low-power, low-latency wireless communication technology^17^. Therefore, it was decided to use ZigBee communication technology to build a wireless network on the construction site.

The ZigBee communication unit is the hardware foundation for ZigBee wireless signal transmission, which can be divided into two categories: low-speed data transmission unit and high-speed data transmission unit. The maximum transmission rate of the low-speed data transmission unit is generally not greater than 150 kB/s, and its carrier has strong penetration and diffraction capabilities, and the transmission distance is relatively long; while the transmission rate of the high-speed data transmission unit can reach more than 500 kB/s, but its carrier The penetrating power and diffraction power are low, and the transmission distance is only tens of meters in open conditions. Due to a large number of obstructions at the construction site of the tower assembly and the large site, the system requires high signal penetration and transmission distance. At the same time, the data collected by the sensor is elementary. The amount of data is counted in bytes, and the amount of data that needs to be transmitted is not large. The requirements for transmission rate are not high. Therefore, combined with the above factors, the system decided to adopt the ZigBee low-speed data transmission unit, and the model adopted the XBee-PRO® 900HP chip^18^.

After the construction data completes the on-site data interaction through the ZigBee communication network, it needs to communicate with the 4G communication unit to realize the long-distance data transmission. Therefore, after completing the hardware selection of the ZigBee communication unit, the 4G communication unit should be selected. The 4G communication unit selects the most mature 4G DTU (Data Transfer Unit), model WH-G405tf chip.

After the system communication module sends out the data, it should be received and displayed by the on-site or remote receiving terminal. Therefore, the on-site human-computer interaction tablet and the remote PC terminal are used as the data receiving terminal. The human-computer interaction tablet selects the PIPO X4 industrial rugged tablet that supports ZigBee communication technology and 4G communication technology access; the remote PC terminal can use a computer that supports USB serial access.

To enable the system to complete data collection, processing and transmission of monitoring points, it is necessary to design and form a complete network transmission scheme. The on-site monitoring adopts a two-way signal transmission method. When data needs to be uploaded, the signal is input from the MCU unit through the DIN pin to the sending end of the ZigBee communication unit and waits for the data to be transmitted. When the receiving end of the ZigBee communication unit receives the data, it is output to the human-computer interaction panel through the DOUT pin. When the human-computer interaction panel needs to issue an instruction, the instruction will enter the sending end of the ZigBee communication unit through the DIN pin. After the receiving end of the ZigBee communication unit receives the signal, the signal will be output to the MCU unit through the DOUT pin to complete the scene. The two-way transmission process of integrated data.

Remote monitoring after the data is received by the human-computer interaction panel, the signal is transmitted to the 4G communication unit and to the 4G base station through the UART (Universal Asynchronous Receiver/Transmitter) serial communication. The signal is transmitted through the mobile communication network, and the data can be accessed through the 4G communication unit, including the State Grid enterprise, remote user platforms such as energy companies and government departments can realize remote monitoring of construction site data.

### System software design

The monitoring system uses formulas to calculate the distance between the monitoring derrick and the tower body in real-time. The formula calculation needs to be realized through program design. Therefore, formula derivation and program design are needed for the distance between the derrick and the tower body.

**Figure 4:**
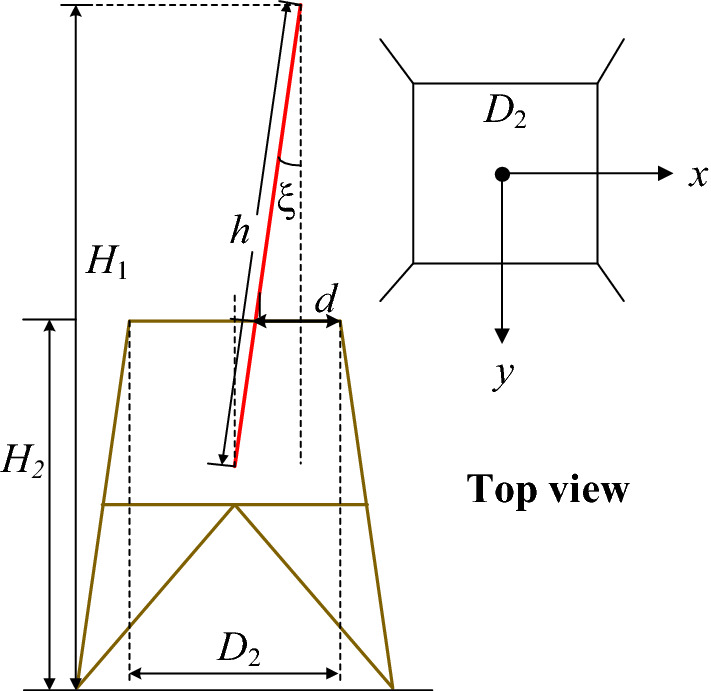
The calculation of the distance between the holding derrick and steel pylon.

Assuming that the distance between the derrick and the tower is *d*, m; the width of the top of the tower is *D*_2_, m; the height of the top of the derrick is *H*_1_, m; the height of the tower is *H*_2_, m; the full length of the derrick is *h*, m; The inclination angle of the derrick is *ξ*. The diagram for calculating the distance between the derrick and the tower body is shown in Fig. 4.

According to the geometric relationship, the formula for calculating the distance *d* between the derrick and the tower body is:

1 $$ d = \frac{{D_{2} }}{2} + (H_{1} - H_{2} )\tan \xi - h\sin \xi . $$

Let the horizontal line direction be the *x*-axis and the forward line direction be the *y*-axis. Since the tilt sensor is a two-axis solid pendulum type, the *x*-axis and *y*-axis of the tilt sensor output are the same as the *x*-axis and *y*-axis in the line direction. The inclination expression can be obtained:

2 $$ \xi { = }\arcsin \left( {\frac{{\sqrt {U_{{\text{x}}}^{2} { + }U_{{\text{y}}}^{2} } }}{{H_{m} g}}} \right), $$

where, *U*_*x*_ and *U*_*y*_ respectively represent the x-axis and y-axis measurement signals output by the dual-axis inclination sensor, V; *H*_m_ represents the output sensitivity of the inclination sensor, V/g; *g* is the acceleration of gravity, m/s^2^.

According to formulas (1) and (2), the distance between the derrick and the tower body can be expressed as:

3 $$ \begin{gathered} d_{x} = \frac{{D_{2} }}{2} + (H_{1} - H_{2} )\tan \left( {\arcsin \left( {\frac{{U_{{\text{x}}} }}{{H_{m} g}}} \right)} \right) - h\sin \left( {\arcsin \left( {\frac{{U_{{\text{x}}} }}{{H_{m} g}}} \right)} \right) \hfill \\ d_{y} = \frac{{D_{2} }}{2} + (H_{1} - H_{2} )\tan \left( {\arcsin \left( {\frac{{U_{y} }}{{H_{m} g}}} \right)} \right) - h\sin \left( {\arcsin \left( {\frac{{U_{y} }}{{H_{m} g}}} \right)} \right), \hfill \\ \end{gathered} $$

where, *d*_x_ and *d*_y_ respectively represent the distance between the derrick and the tower in the horizontal direction and the distance between the derrick and the tower in the direction of the line, m.

After clarifying the formula for calculating the distance between the derrick and the tower, it is necessary to design the program for the distance between the derrick and the tower. First, according to the construction engineering parameters of the tower, combined with the construction design drawings to obtain the full-length *h* of the derrick. After the construction of the tower is started, according to the real-time construction progress, combined with the tower design drawings and construction plan to obtain the tower body width *D*_2_ and the tower height *H*_2_ (for example, when the tower is assembled to 13 m, the tower body width *D*_2_ = 4.5 m, the height of the tower *H*_2_ = 13 m; when the tower is assembled to 20 m, the width of the tower is *D*_2_ = 3.8 m, and the height of the tower *H*_2_ = 20 m).

At the same time, according to the real-time monitoring data of the derrick-holding angle and height sensor, the real-time derrick-holding angle *ξ* and the height of the top of the derrick-holding *H*_1_ are obtained. After obtaining the above data, the distance between the derrick and the tower body *d*_x_ and *d*_y_ can be finally obtained according to the derived calculation formula for the distance between the derrick and the tower body, and the entire program operation process of the distance between the derrick and the tower body is completed.

The design of the field data transmission program is the key to realize the system data transmission. The design of the early warning program directly determines the effect of the system on the safety monitoring of the tower construction.

In view of the characteristics of the system with many monitoring points and wide distribution, the field data transmission and early warning procedure adopt the polling mode, each monitoring point in different addresses of the system is asked separately, and the next monitoring is started after one monitoring point answers. Point inquiries, and in this way, the cycle is repeated to achieve the orderly use of each channel through time division.

In the process of data transmission, the monitoring terminal first starts to call the target address code and judges. If the address code does not match, the program will be interrupted and return to the start state; if the target address code matches the local address code, the address data will be sent (a data acquisition instruction is issued) And wait for the data to return, if the sending fails, the command will be sent again until it succeeds.

After the address data transmission is completed, the monitoring terminal waits for the data return reception. If the data reception is unsuccessful for more than three times, it is judged as a communication failure. At this time, the next target address code will be called immediately; if the data return reception is successful, the monitoring. The terminal analyzes and calculates the received data and clears the number of unsuccessful data receptions.

The remote data transmission program design directly determines the effect of the system’s remote monitoring function, so it is necessary to design the remote data transmission program.

The remote transmission of the system adopts the network transparent transmission mode without protocol encapsulation, which is directly sent to the network server through the serial port of the module without any processing or modification. In the network transparent transmission mode, the human-computer interactive tablet uses the sending end of the G405tf communication module to package and send data to the receiving end of the remote G405tf communication module through the public 4G communication network and through the “transparent cloud” platform to achieve data interconnection. Real-time forwarding to the remote PC end completes the entire remote data transmission process.

## Results

**Figure 5:**
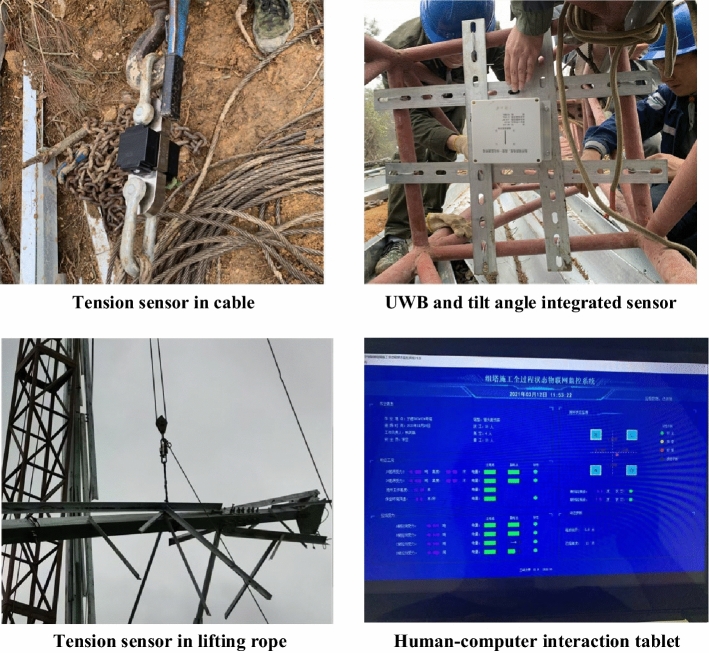
The layout of the monitoring system on the project site.

After the development of the monitoring system is completed, it is necessary to verify the accuracy of the monitoring data of the system and the delay of data transmission. This system is applied in the 500 kV transmission project of Fujian Zhouning Pumped Storage Power Station. The site layout is shown in Fig. 5.

The accuracy of system monitoring data directly determines the system’s early warning effect. Take a base tower in the 500 kV transmission project of Fujian Zhouning Pumped-storage Power Station as an example. The tower type is 5B1-ZBC1K, the total height of the derrick is 29 m, the mass of the ninth section of the lifting tower is 2056.75 kg, and the mass of the rigging is 125.4 kg. Table 2 shows the comparison of the actual measured value and system monitoring value at a certain moment.

**Table 2:** Comparison of measured data and calculated data of the system.

Parameter	Actual value	Monitoring value	Relative error/%
Cable tension	5.85 kN	6.02 kN	2.91
Lifting rope tension	21.34 kN	20.47 kN	− 4.08
Derrick inclination	4.7°	4.9°	4.26
Derrick height	38.48 m	40.1 m	4.21
Tower material height	18.48 m	19.89 m	7.63
The distance between the derrick and the assembled tower	3.24 m	3.56 m	9.88

The inclination, height, and distance data in Table 2 “Actual Values” are accurately measured by Leica TS50 total station, and the tensile force data is calculated according to the calculation model given in the construction plan, combined with the actual measured inclination, distance and other parameters. It can be seen that the minimum monitoring error of the pull wire tension is 2.91%. The monitoring errors of the height of the hanger and the distance between the pole and the tower body are relatively large, which are 7.63% and 9.88%, respectively. It can be seen from the analysis that the monitoring error of the hanging piece height mainly comes from the influence of the temperature and humidity changes on the construction site on the data collection of the air pressure height sensor, resulting in a certain error in the monitoring of the hanging piece height. The monitoring error of the distance between the pole and the tower body is mainly due to the fact that the suspension pole is not completely located in the center of the tower during the construction process, which leads to the misjudgment of the distance between the pole and the tower body in the calculation result of the formula, resulting in a certain error in the monitoring value. But on the whole, the actual measured values of the above parameters match well with the system monitoring values, with an average error of 4.14%. Because the error mainly comes from the on-site environment and the irregular operation of the construction personnel, rather than the system’s error. Therefore, the system monitoring data can well perceive the attitude information during the construction of the pole. It can be seen that the monitoring system of the suspension pole group tower is well applied in the field, and the performance in all aspects can meet the engineering requirements.

The timeliness of the data transmission of the monitoring system directly affects its monitoring effect. The lower the data transmission delay of the system, the faster the construction personnel can adjust the construction risk points. To measure the data transmission delay of the system, we use the commonly used data transmission delay measurement system ping (Packet Internet Groper) to obtain the data transmission delay of this system. Because the data transmission delay of the system is affected by environmental factors, we measured the on-site and remote data transmission delays of the system at five different locations. The measurement conditions are shown in Table 3. The system’s on-site average data latency is 18 ms, and the long-range average data transfer latency is 176 ms. Compared with the data transmission delay of monitoring systems in other fields, it can be seen that the data transmission delay of the system on-site and remote is relatively low, and it has good data transmission timeliness.

**Table 3:** Data transfer delay test situation.

	Construction site data transmission delay	Remote data transfer delay
Location 1	15 ms	162 ms
Location 2	18 ms	174 ms
Location 3	22 ms	187 ms
Location 4	20 ms	180 ms
Location 5	15 ms	177 ms
Average value	18 ms	176 ms

## Conclusion

In view of the unsafe factors existing in the construction process of the suspended pole tower, this paper develops a monitoring system for the suspended pole tower based on the Internet of Things, which realizes all-round intelligent perception and danger early warning of the tower construction, and improves the safety of the group tower project. The system has been verified by the 500 kV delivery project of Fujian Zhouning Pumped Storage Power Station with good application. The average data transmission delays in the far and near fields of the system are 28 ms and 156 ms, and the average error of key monitoring point data is 4.14%. The results show that the system has a good effect on monitoring the construction of suspended pole-mounted towers. The average error of key monitoring point data is 4.14%, and the monitoring data is accurate.

## Data Availability

All data generated or analysed during this study are included in this published article.
